# Supplementary material for: Public perceptions and support of climate intervention technologies across the Global North and Global South
Source: Nat Commun. 2024 Mar 6;15:2060. doi: 10.1038/s41467-024-46341-5 (PMC10918186; doi:10.1038/s41467-024-46341-5)
Supplement: Supplementary file 1 — Supplementary Information [file 41467_2024_46341_MOESM1_ESM.pdf]

Supplementary Tables and Figures

Supplementary Table 1: List of countries and language in which survey was conducted

Country	Language
United States	English, Spanish
United Kingdom	English
Australia	English
Canada	English, French
Austria	German
Germany	German
France	French
Switzerland	German, French, Italian
Sweden	Swedish
Denmark	Danish
Norway	Norwegian
Estonia	Estonian
Poland	Polish
Greece	Greek
Italy	Italian
Netherlands	Dutch
Spain	Spanish
Chile*	Spanish
Dominican Republic*	Spanish
Brazil*	Portuguese
Japan	Japanese
China*	Chinese Simplified
Singapore*	Chinese Simplified, English
Indonesia*	Indonesian
India*	Hindi, English
Turkey	Turkish
South Africa*	English
Kenya*	English
Nigeria*	English
Saudi Arabia*	Arabic, English

Note: Asterisks are used to denote countries in the Global South cohort.

Supplementary Table 2: Overview of Survey Design

Procedure	Related measures
Screening questions	Age, Gender, Geographic area, Region
Introductory text on CDR and SRM	
Randomized assignment to technology grouping:<br>(1) Solar Radiation Management; (2) Carbon Dioxide Removal 1 (ecosystem-based); (3) Carbon Dioxide Removal 2 (engineered)	
Comprehension questions – 2 items	
Familiarity with technologies	*Familiarity* – 1 item per technology
Assessment of perceived risks/benefits	*Perceived risks* – 4 items per technology<br>*Perceived benefits* – 4 items per technology (adapted from Jobin and Siegrist (2020), based on Wright et al. (2014))
Ranking of risks	*Risk ranking* – ranking of up to 4 risks (for SRM) and 5 risks (for CDR1, CDR2)
Weighing risks and benefits	*Risk-benefit weight* – 1 item per technology (adapted from Pidgeon and Spence 2017)
Assessments of support for technology	*Perceived support* – 3 items per technology (1 on research and development, 1 on small-scale field trials, 1 on broad deployment), adapted from Pidgeon and Spence (2017) and Jobin and Siegrist (2020)
Assessment of policy support	*Policy support* – choice of up to seven policies at international or domestic level
Assessment of potential covariates	*Aversion to tampering with nature* – 5-item scale, developed by Wolske et al. (2019)<br>*Environmental identity* – 3-item measure adapted from van de Werff et al. (2013)<br>*Trust in institutions and science* – 5-item measure, adapted from Jobin and Siegrist (2020)<br>*Sources of information* – choice of up to nine information sources as credible<br>*Affect related to climate change* – 4 items, adapted from Feldman and Hart (2021)<br>*Concern over climate change* – 3 items, adapted from Steentjes et al. (2017)<br>*Beliefs about climate change* – 2 items<br>*Ownership of green products* – 2 items (1 on solar panels, 1 on electric vehicles
Demographics	Occupation, Education, Income, Religiosity, Political views, Member of minority or indigenous group
End Question and Debrief	

Supplementary Figure 1: Background Information

The negative effects of climate change are becoming more apparent.

Caused by the human-driven release of carbon dioxide (CO2) and other greenhouse gases, the Earth's temperature is increasing, sea levels are rising, and extreme weather events are happening more often.

Measures to help limit the effects of climate change have been proposed, including reducing how much greenhouse gas is emitted (mitigation) and preparing for the current and predicted impacts of climate change (adaptation).

The current survey focuses on two more suggestions: carbon dioxide removal and solar radiation management. The first aims to remove CO2 from the atmosphere, before storing in plants, underground, or at the bottom of the ocean. The second aims to reflect how much sunlight reaches the Earth in order to reduce global temperature levels.

In the rest of the survey, we would like to know what you think about a few of these measures.

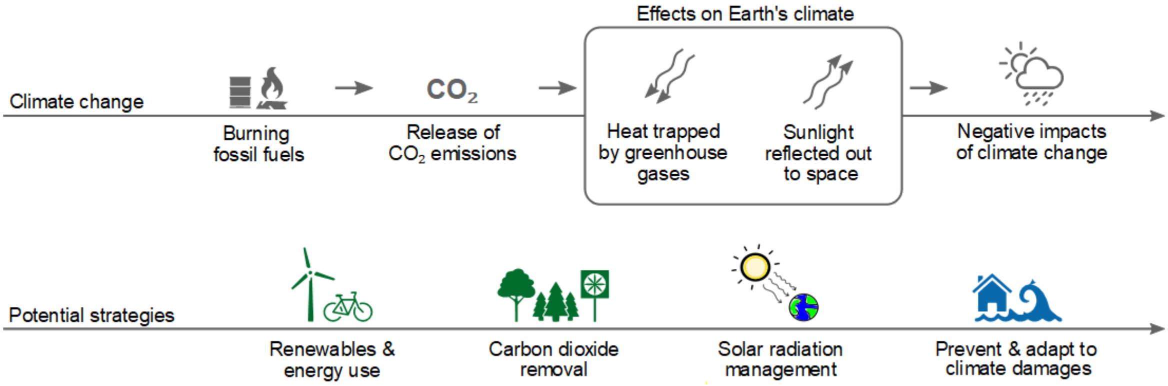

Note: Credit for the source graphic, from which this Figure has been adapted, to William Lamb and the Mercator Research Institute on Global Commons and Climate Change (MCC).

Supplementary Figure 2: Information Texts for all Climate-Intervention Technologies

We will now provide you with some background information on a few technologies. In the remainder of the survey, we want to get your feedback on these technologies, so please read the texts carefully.

To make sure you have understood them, the next slide will have one or two short questions to see how well you have understood. In order that you do not feel the need to rush, you will only be able to click to the next slide after 15 seconds have passed.

Group 1 (SRM)

Stratospheric Aerosol Injection – This aims to limit the effects of climate change by using planes or balloons to spray small particles (aerosols) into the upper atmosphere.

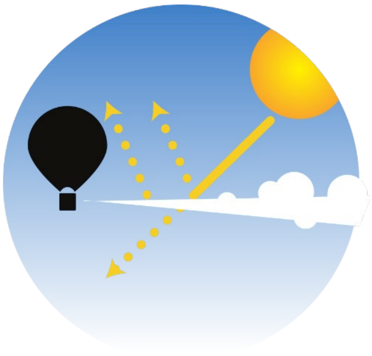

The particles would reflect sunlight back into space. This could cool temperatures on Earth. But for this idea to work, we would have to keep doing it continuously. If we stopped, temperatures would rise once again, and probably very quickly. This would not do anything to reduce our greenhouse gas emissions or help with other impacts, such as ocean acidification.

Marine Cloud Brightening –

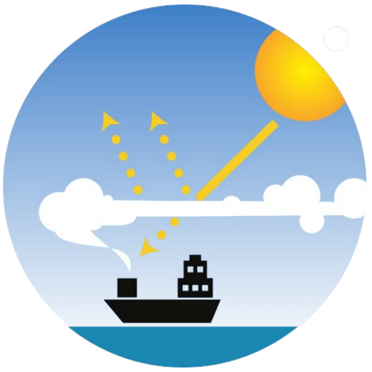

This aims to limit the effects of climate change by spraying small particles, such as sea salt, into the air over the oceans, to make clouds brighter.

These clouds would reflect sunlight away from the Earth, which could cool temperatures on a local or regional level. Marine cloud brightening might also help to protect ecosystems threatened by climate change. One way to spray the particles would be to use a fleet of ships. But for it to work, we would have to keep doing it continuously. If we stopped, temperatures would rise once again. Also, marine cloud brightening would not do anything to reduce greenhouse gas emissions or help with other impacts, such as ocean acidification.

Space-based Geoengineering –

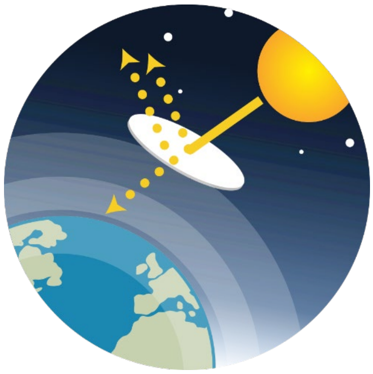

This aims to limit the effects of climate change by putting a giant mirror or other reflective material in outer space between the Earth and the sun.

Such a space mirror or sunshield would deflect sunlight back into space. This could cool temperatures on Earth. This would avoid direct changes to the land, oceans, or atmosphere of the Earth itself. But for this idea to work, we would need to be able to build and maintain something in space much larger than ever before. A space mirror would also be very costly to build, given that its location would be about four times as far from the Earth as the Moon. Also, it would not do anything to reduce our greenhouse gas emissions or help with other impacts, such as ocean acidification.

Group 2 (CDR1)

Afforestation and Reforestation *–*

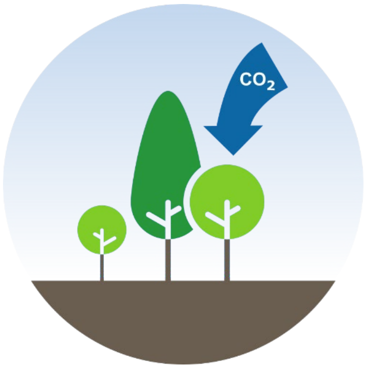

Both aim to limit the effects of climate change by planting trees.

As trees grow, they absorb carbon dioxide from the atmosphere and store it for decades or longer, as long as the forest is around. But for this idea to work, we would need a lot of land and water. It is therefore likely to compete with agriculture and other uses. Also, if trees were cut down or happen to burn down, then the carbon dioxide would again be released into the atmosphere.

Soil Carbon Sequestration *–*

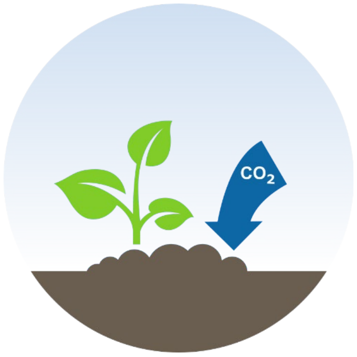

This aims to limit the effects of climate change by changing agricultural techniques to store more carbon dioxide in soils.

This could include planting different crops, leaving crop residues on the field, or increasing the number of trees on agricultural lands. By improving soils, it is possible to remove carbon dioxide from the air and store it in soils for decades to centuries. Soil carbon sequestration also requires no additional land and might make soils better for farming. But for it to work, we would need farmers and people in other industries to cooperate and take part. Also, if agricultural practices are not sustained, the carbon dioxide would again be released into the atmosphere.

Marine Biomass and Blue Carbon *–*

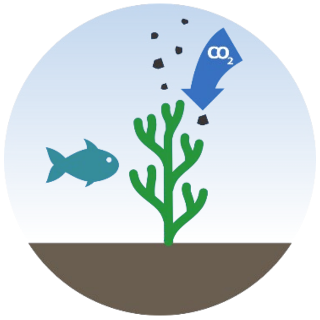

Both aim to limit the effects of climate change by improving how much carbon dioxide is stored in the oceans.

Blue carbon does this by restoring or growing ecosystems such as mangroves, salt marshes, and seagrass meadows. Marine biomass does this by growing seaweeds or macroalgae. All of these absorb carbon dioxide from the atmosphere as they grow. This can then be stored for decades to centuries at the bottom of the ocean. But for this idea to work, we would need many people, especially those in coastal communities, to cooperate and take part. Also, if ecosystems are disturbed or destroyed or the plants are cut down, the carbon dioxide would again be released into the atmosphere.

Group 3 (CDR2)

Group 3 (CDR2)

Direct Air Capture with Carbon Storage *–*

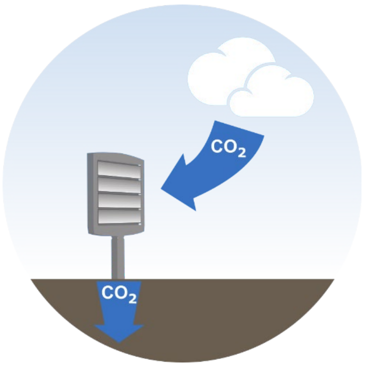

This aims to limit the effects of climate change by using very large fans to remove carbon dioxide from the air (direct air capture).

Once pulled into the fans, absorptive liquids convert the carbon dioxide using a chemical process. It can then be stored indefinitely underground (carbon storage). Direct air capture with carbon storage also needs little land. But for it to work, it would require lots of energy along with underground places to store carbon. Direct air capture is also extremely expensive right now and it is not clear if it works at the large scales needed, both of which limit how much it can be used.

Bioenergy with Carbon Capture and Storage *–*

This aims to limit the effects of climate change by growing and harvesting plants as a source of energy (bioenergy).

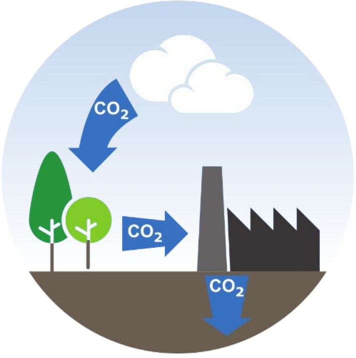

As plants grow, they absorb carbon dioxide from the air. By burning these plants and chemically capturing the carbon dioxide released, bioenergy can provide energy for homes and businesses or be stored underground indefinitely (carbon capture and storage). But for it to work, we would need a lot of land and water (and underground places to store carbon). It is therefore likely to compete with agriculture and other uses. It is also not clear if bioenergy with carbon capture and storage will work at the large scales needed, though some industrial applications do already exist.

Enhanced Weathering *–*

This aims to limit the effects of climate change by increasing the ability of rocks to absorb carbon dioxide from the atmosphere.

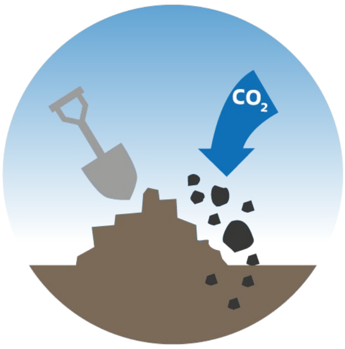

As rocks such as limestone and basalt are exposed in nature to processes like rain, wind, or waves, they are ground down (weathering), which allows them to absorb carbon dioxide. Since this process takes place extremely slowly, enhanced weathering speeds it up by physically or chemically grinding the rocks before placing them onto soils, beaches, or next to rivers. Over time, rocks and their carbon dioxide are ultimately stored in oceans indefinitely. But for it to work, we would need a lot of rocks. This could cause negative ecological and human health impacts (and greater energy use) from more mining and extraction. Also, it is not clear if it will work at the large scales needed, as only limited trials have been done so far.

Biochar –

This aims to limit the effects of climate change by heating organic material, such as tree branches and cornstalks, inside a container with no oxygen.

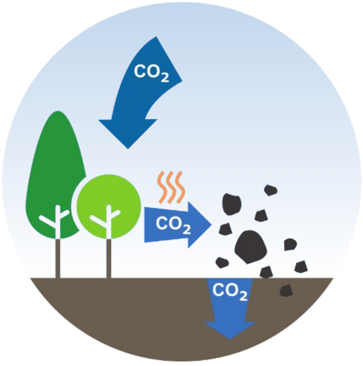

This creates black material very similar to charcoal (biochar). If we grind this up and add it to soil, it is possible to remove carbon dioxide from the air and store it in soils for decades to centuries. Biochar might also make better soils for farming. It could also be added to other things, such as concrete, animal feed, or compost. But for it to work, we would need farmers and people in other industries to take part and, potentially, change how they do things. We would also need a lot of organic material. Biochar is also quite expensive right now, which limits how much it can be used.

Note: We are grateful for and acknowledge William Lamb and the Mercator Research Institute on Global Commons and Climate Change (MCC) and their assistance with designing and providing funding for the icon graphics for each of the technologies.

Statistical Analysis of Significant Differences between Technology Categories regarding Perceptions of Risks and Benefits and Level of Support for Policy Options

Though not included in the main text, pairwise comparisons were also employed to assess if significant differences existed between the technology categories in relation to perceptions of risks and benefits and support for policy options. We report the details on the results and test statistics here for any interested readers.

Perceptions of Risks and Benefits

Nonparametric testing in the form of the pairwise, independent-samples (two-tailed) Kruskal-Wallis *H* test was used to identify significant differences (and the lack thereof) both at the level of technology category (i.e., solar radiation modification (SRM) versus ecosystem-based carbon dioxide removal (nCDR) versus engineered carbon dioxide removal (eCDR)) and between Global North and Global South cohorts. At a general level (for the global sample), we identify significant differences in perceived risks/benefits between technology categories: for "can be done safely in a controlled fashion", $\chi2(2) = 136.662$, $p = .000$; for "cost-efficient and cheaper than cutting use of fossil fuels", $\chi2(2) = 159.084$, $p = .000$; for "environmentally friendly", $\chi2(2) = 167.608$, $p = .000$; for "can be counted on in the long term", $\chi2(2) = 173.522$, $p = .000$; for "leads to unintended side effects", $\chi2(2) = 132.990$, $p = .000$; for "would distribute risks unequally between rich and poor countries", $\chi2(2) = 39.383$, $p < .001$; for "threat to humans and nature", $\chi2(2) = 119.362$, $p = .000$; and for "would decrease the motivation to reduce CO2 emissions", $\chi2(2) = 10.955$, $p = .004$.

Conducting post hoc testing of pairwise comparisons (Supplementary Table 3), we identified, through independent-samples (two-tailed) Kruskal-Wallis *H* testing, with significance values adjusted by the Bonferroni correction for multiple tests, specific risks and benefits where perceptions significantly differed between pairs of technology categories. We find that all technology categories differed significantly for "can be done safely in a controlled fashion": for SRM versus eCDR, $H = -50.936$, $Z=-4.211$, $p =.000$; for SRM versus nCDR, $H = -148.289$, $Z=-11.468$, $p =.000$; for eCDR versus nCDR, $H = 97.353$, $Z=8.048$, $p =.000$. Significant differences were also identified for "cost-efficient and cheaper than cutting use of fossil fuels": for SRM versus eCDR, $H = -46.715$, $Z=-3.862$, $p =.000$; for SRM versus nCDR, $H = -157.917$, $Z=-12.212$, $p =.000$; for eCDR versus nCDR, $H = 111.201$, $Z=9.193$, $p =.000$; as well as for "environmentally friendly": for SRM versus eCDR, $H = -43.761$, $Z=-3.618$, $p =.001$; for SRM versus nCDR, $H = -160.856$, $Z=-12.931$, $p =.000$; for eCDR versus nCDR, $H = 117.941$, $Z=9.681$, $p =.000$; and for "can be counted on in the long term": for SRM versus eCDR, $H = -66.344$, $Z=-5.485$, $p =.000$; or SRM versus nCDR, $H = -168.800$, $Z=-13.054$, $p =.000$; and for eCDR versus nCDR, $H = 102.456$, $Z=8.470$, $p =.000$. Regading risks, perceptions that technology categories might "lead to unintended side effects" were significantly different: for SRM versus eCDR, $H = -38.126$, $Z=-3.152$, $p =.005$; for SRM versus nCDR, $H = -143.017$, $Z=-11.060$, $p =.000$; for eCDR versus nCDR, $H = 104.890$, $Z=8.672$, $p =.000$; as well as that they "would distribute risks unequally between rich and poor countries: for SRM versus eCDR, $H = -43.108$, $Z=-3.564$, $p =.001$; for SRM versus nCDR, $H = 31.811$, $Z=2.460$, $p =.042$; for eCDR versus nCDR, $H = -74.919$, $Z=-6.194$, $p =.000$. Regarding perceptions of the "threat to humans and nature", while ecosystem-based CDR was significantly different from SRM, $H = 128.067$, $Z=9.094$, $p =.000$, and from engineered CDR, $H = -111.186$, $Z=-9.192$, $p =.000$, this was not true of SRM and engineered

CDR, *H* = 16.881, *Z*=1.396, *p* =.163. While perceptions of engineered CDR and ecosystem-based CDR were significantly different for "would decrease the motivation to reduce CO2 emissions, *H* = -39.172, *Z*=-3.239, *p* =.004, this was not true for SRM vis-à-vis ecosystem-based CDR, *H* = 29.844, *Z*=2.308, *p* =.063, or SRM vis-à-vis engineered CDR, *H* = -9.328, *Z*=-0.771, *p* =1.000.

Supplementary Table 3: Global-level perceptions of risks and benefits, by technology category (different letters in columns indicate significant differences in mean level of perceived risks/benefits of different technology categories;1-7 scale: 1= Strongly disagree, 4=Neither agree nor disagree, 7 = Strongly agree)

	Solar Radiation Modification	Engineered Carbon Dioxide Removal	Ecosystem-based Carbon Dioxide Removal
Benefits			
Can be done safely in a controlled fashion	4.35[a]	4.77[b]	5.39[c]
Cost-efficient and cheaper than cutting use of fossil fuels	3.67[a]	4.09[b]	4.96[c]
Environmentally friendly	4.34[a]	4.71[b]	5.58[c]
Can be counted on in the long term	4.03[a]	4.59[b]	5.32[c]
Risks			
Leads to unintended side effects	4.33[a]	4.17[b]	3.57[c]
Would distribute risks unequally between rich and poor countries	4.10[a]	4.22[b]	3.97[c]
Threat to humans and nature	3.87[a]	3.79[a]	2.99[b]
Would decrease the motivation to reduce CO2 emissions	4.29[a]	4.33[a]	4.17[b]

Note: N=30,284 participants. If different letters present for columns of given row ("a" versus "b"), this indicates that significant differences exist among perceived benefits/risks of the respective technology categories ($p < 0.05$), according to pairwise, independent-samples (two-tailed) Kruskal-Wallis *H* testing, with adjustments for multiple comparisons using the stepwise step-down method (for technologies). If the letters are the same (i.e., "a" appears throughout), no significant differences exist. Specific details on test statistics and significant testing can be found in the Methods and Survey Description section. Means (rather than the mean ranks, on which the tests are based) are reported. Items employed 1-7 scale: 1= Strongly disagree, 4=Neither agree nor disagree, 7 = Strongly agree. Cells are colored according to following scheme: for benefits items, those just above the midpoint from 4.00-4.49 are shaded in pale yellow, those from 4.50-4.99 pale green, those from 5.00-5.49 slightly darker green, and those from 5.50-6.00 rather dark green. Conversely, cells below the midpoint from 3.50-3.99 are shaded pale orange. The shading scheme is inverted for risk items since agreement here signifies a stronger sense of risks being present. The overall pattern for a particular technology can thus be discerned by reading thing as one might a traffic light.

As represented in Supplementary Table 4, nonparametric testing in the form of the pairwise, independent-samples (two-tailed) Kruskal-Wallis *H* test was also used to identify significant differences (and the lack thereof) between the technology categories for each cohort (Global North and Global South). Starting with the Global North, we find that all technology categories differed significantly for "can be done safely in a controlled fashion": for SRM versus eCDR, *H* = -47.099, *Z*=-4.888, *p* =.000; for SRM versus nCDR, *H* = -112.202, *Z*=-10.893, *p*=.000; for eCDR vs. nCDR, *H* = 65.103, *Z*=6.757, *p*=.000. Significant differences were also identified for "cost-efficient and cheaper than cutting use of fossil fuels": for SRM versus eCDR, *H* = -33.033, *Z*=-3.428, *p* =.002; for SRM versus nCDR, *H* = -109.728, *Z*=-10.652, *p* =.000; for eCDR versus nCDR, *H* = 76.695, *Z*=7.960, *p* =.000; as well as for

"environmentally friendly": for SRM versus eCDR, *H* = -37.564, *Z*=-3.899, *p* =.000; for SRM versus nCDR, *H* = -113.132, *Z*=-10.983, *p* =.000; for eCDR versus nCDR, *H* = 75.568, *Z*=7.843, *p* =.000; and for "can be counted on in the long term": for SRM versus eCDR, *H* = -49.059, *Z*=-5.092, *p* =.000; or SRM versus nCDR, *H* = -118.447, *Z*=-11.499, *p* =.000; and for eCDR versus nCDR, *H* = 69.388, *Z*=7.201, *p* =.000. On the risks side, perceptions that technology types might "lead to unintended side effects" were significantly different: for SRM versus eCDR, *H* = 32.884, *Z*=3.413, *p* =.002; for SRM versus nCDR, *H* = 107.412, *Z*=10.428, *p* =.000; for eCDR versus nCDR, *H* =-74.529, *Z*=-7.735, *p* =.000. However, while perceptions that engineered CDR "would distribute risks unequally between rich and poor countries" were significantly different vis-à-vis SRM, *H* = -42.803, *Z*=-4.443, *p* =.000, and ecosystem-based CDR, *H* = -54.171, *Z*=-5.623, *p* =.000, there was no significant difference between SRM and ecosystem-based CDR, *H* = 11.368, *Z*=1.104, *p* =.809. Regarding perceptions of the "threat to humans and nature", while ecosystem-based CDR was significantly different from SRM, *H* = 98.404, *Z*=9.553, *p* =.000, and from engineered CDR, *H* = -77.864, *Z*=-8.081, *p* =.000, this was not true of SRM and engineered CDR, *H* = 20.539, *Z*=2.132, *p* =.099. Lastly, while perceptions of SRM and ecosystem-based CDR were significantly different on if they "would decrease the motivation to reduce CO2 emissions, *H* = 37.088, *Z*=3.601, *p* =.001, this was not true for SRM vis-à-vis engineered CDR, *H*= 14.224, *Z*=1.476, *p* =.420, or engineered vis-à-vis ecosystem-based CDR, *H* = -22.864, *Z*=-2.373, *p* =.053.

Supplementary Table 4: Global North versus Global South perceptions of risks and benefits, according to technology category (different letters in columns indicate significant differences in mean perceptions of risks/benefits of different technology categories; different numbers in "Global North" and "Global South" Tables (1 versus 2) indicate significant differences of Global North versus Global South; 1-7 scale: 1= Strongly disagree, 4=Neither agree nor disagree, 7 = Strongly agree)

**Global North**	Solar Radiation Modification	Engineered Carbon Dioxide Removal	Ecosystem-based Carbon Dioxide Removal
Benefits			
Can be done safely in a controlled fashion	4.04[1,a]	5.26[1,b]	4.56[1,c]
Cost-efficient and cheaper than cutting use of fossil fuels	3.43[1,a]	4.80[1,b]	3.86[1,c]
Environmentally friendly	4.06[1,a]	5.49[1,b]	4.49[1,c]
Can be counted on in the long term	3.78[1,a]	5.21[1,b]	4.38[1,c]
Risks			
Leads to unintended side effects	4.38[1,a]	3.49[1,b]	4.18[1,c]
Would distribute risks unequally between rich and poor countries	4.02[1,a]	3.97[1,a]	4.19[1,b]
Threat to humans and nature	3.90[1,a]	2.87[1,b]	3.77[1,a]
Would decrease the motivation to reduce CO2 emissions	4.22[1,a]	4.02[1,b]	4.16[1,ab]

**Global South**	Solar Radiation Modification	Engineered Carbon Dioxide Removal	Ecosystem-based Carbon Dioxide Removal
Benefits			
Can be done safely in a controlled fashion	4.88[2,a]	5.62[2,b]	5.12[2,c]
Cost-efficient and cheaper than cutting use of fossil fuels	4.07[2,a]	5.22[2,b]	4.49[2,c]
Environmentally friendly	4.83[2,a]	5.73[2,b]	5.09[2,c]
Can be counted on in the long term	4.47[2,a]	5.51[2,b]	4.96[2,c]
Risks			
Leads to unintended side effects	4.25[2,a]	3.70[1,b]	4.14[1,a]
Would distribute risks unequally between rich and poor countries	4.23[2,a]	3.97[1,b]	4.27[1,a]
Threat to humans and nature	3.81[2,a]	3.20[2,b]	3.81[1,a]
Would decrease the motivation to reduce CO2 emissions	4.40[2,a]	4.43[2,a]	4.64[2,b]

Note: N=30,284 participants. If different letters present for columns of given row "a" versus "b"), this indicates that significant differences exist among perceived benefits/risks of the respective technology categories ($p < 0.05$), according to pairwise, independent-samples (two-tailed) Kruskal-Wallis H testing, with significance values adjusted by Bonferroni correction for multiple tests. If letters are the same (i.e., "a" appears throughout), no significant differences exist. If two letters appear (e.g., "ab"), as for "Engineered Carbon Dioxide Removal" in row "would decrease the motivation to reduce CO2 emissions", this indicates that perceptions do not significantly differ from the either two technology categories, though these differ from one another. If the numbers are different between "Global North" and "Global South" tables (i.e., "2" appears in corresponding cell for "Global South"), this indicates significant differences exist between perceptions of these cohorts, according to independent-samples (two-tailed) Mann-Whitney U testing. Specific details on the test statistics and significant testing can be found in the Methods and Survey Description section Means (rather than mean ranks, on which the tests are based) are reported. Items employed 1-7 scale: 1= Strongly disagree, 4=Neither agree nor disagree, 7 = Strongly agree. Cells are colored according to the following scheme: for benefits items, those above the midpoint from 4.00-4.49 are shaded pale yellow, those from 4.50-4.99 pale green, those from 5.00-5.49 darker green, and those from 5.50-6.00 quite dark green. Conversely, cells below the midpoint from 3.50-3.99 are pale orange, those between 3.00 and 3.49 orangish-red. The shading scheme is inverted for risk items, as agreement here signifies a stronger sense of risks being present. Cells above the midpoint from 4.00-4.49 are pale orange here whereas those just below are pale yellow. As values decrease away from the mid-point, they become increasingly green in shading.

Turning to the Global South cohort, we find all technology categories differed significantly for "can be done safely in a controlled fashion": for SRM versus eCDR, H = -19.686, Z=-2.680, p =.022; for SRM versus nCDR, H = -59.409, Z=-7.566, p =.000; for eCDR versus nCDR, H = 39.723, Z=5.408, p =.000. Significant differences were also identified for "cost-efficient and cheaper than cutting use of fossil fuels": for SRM versus eCDR, H = -19.087, Z=-2.599, p =.028; for SRM versus nCDR, H = -60.864, Z=-7.751, p =.000; for eCDR versus nCDR, H = 41.777, Z=5.688, p =.000; as well as for "environmentally friendly": for SRM versus eCDR, H = -17.932, Z=-2.442, p =.044; for SRM versus nCDR, H = -60.333, Z=-7.684, p =.000; for eCDR versus nCDR, H = 42.402, Z=5.773, p =.000; and for "can be counted on in the long term": for SRM versus eCDR, H = -27.977, Z=-3.809, p =.000; or SRM versus nCDR, H = -64.061, Z=-8.158, p =.000; and for eCDR versus nCDR, H = 36.083, Z=4.913, p =.000. On the risks side, we identified no significant difference between engineered CDR and SRM when it comes to perceptions that these could "lead to unintended side effects", H = 7.220, Z=0.983, p =.977, "would distribute risks unequally between rich and poor countries", H = -3.527, Z=-0.480, p =1.000, and are a "threat to humans and

nature", $H = 0.208$, $Z{=}0.280$, $p = 1.000$. In contrast, there were significant differences for "lead to unintended side effects" between ecosystem-based CDR and SRM, $H = 37.697$, $Z{=}4.801$, $p = .000$, and ecosystem-based CDR and engineered CDR, $H = -30.477$, $Z{=}-4.149$, $p = .000$; for "would distribute risks unequally between rich and poor countries" between ecosystem-based CDR and SRM, $H = 19.348$, $Z{=}2.464$, $p = .041$, and ecosystem-based CDR and engineered CDR, $H = -22.875$, $Z{=}-3.114$, $p = .006$; as well as for "threat to humans and nature" between ecosystem-based CDR and SRM, $H = 33.106$, $Z{=}4.216$, $p = .000$, and ecosystem-based CDR and engineered CDR, $H = -32.898$, $Z{=}-4.479$, $p = .000$. On the other hand, while perceptions that engineered CDR "would decrease the motivation to reduce CO2 emissions" were significantly different from those for SRM, $H = -20.045$, $Z{=}-2.729$, $p = .019$, and ecosystem-based CDR, $H = -19.803$, $Z{=}-2.696$, $p = .021$, this was not true for SRM vis-à-vis ecosystem-based CDR, $H = -0.242$, $Z{=}-0.031$, $p = 1.000$.

Level of Support for Policy Options

Nonparametric testing in the form of the pairwise, independent-samples (two-tailed) Kruskal-Wallis *H* test, with significance values adjusted by the Bonferroni correction for multiple tests, was used to identify significant differences in policy support (and lack thereof) at the level of technology category (solar radiation modification (SRM) versus ecosystem-based carbon dioxide removal versus engineered carbon dioxide removal) for Global North and Global South cohorts. Starting with the Global North, as represented in Supplementary Table 5a, we find policy support does not significantly differ across the technology categories for "independent national government policies restricting use of technology", $\chi2(2) = 1.664$, $p{=}.435$, "information and engagement campaigns to consult and inform public", $\chi2(2) = 4.716$, $p = .095$, "international ban or moratorium on technologies deemed risky", $\chi2(2) = 0.837$, $p = .658$, "global-level market for trading carbon credits and/or offsets", $\chi2(2) = 1.263$, $p = .261$, and "none of the above, $\chi2(2) = 4.753$, $p = .093$. In contrast, there are significant differences between the technology types in terms of policy support for "national-level support and funding by governments to enable technology for public and private research and development", $\chi2(2) = 27.769$, $p < .001$, "establishment of international organization to conduct oversight and set standards", $\chi2(2) = 17.025$, $p < .001$, and "generating special report at international level (e.g., by IPCC) to evaluate and assess technologies", $\chi2(2) = 6.865$, $p = .032$. Looking at pairwise comparisons for the three policies, support for "national-level support and funding by governments to enable technology for public and private research and development" is significantly lower for SRM vis-à-vis ecosystem-based CDR, $H = -28.368$, $Z = -5.268$, $p = .000$, and engineered CDR, $H = -14.737$, $Z = -2.737$, $p{=}.019$, while support for ecosystem-based CDR is statistically higher than engineered CDR, $H = 13.632$, $Z{=}2.532$, $p = .034$. Regarding support for "establishment of international organization to conduct oversight and set standards", this is significantly lower for SRM versus ecosystem-based CDR, $H = -21.921$, $Z = -4.072$, $p = .000$, and engineered CDR, $H = -14.079$, $Z = -2.615$, $p{=}.027$, while support for the two types of CDR does not differ, $H = 7.842$, $Z{=}1.457$, $p = .145$. However, for "generating special report at international level (e.g., by IPCC) to evaluate and assess technologies", there is a discrepancy between the significance findings, ostensibly due to the conservative tendencies of Bonferroni correction (Armstrong 2014; Rothman 1990). Resorting instead to independent-samples Mann-Whitney *U* testing, we find that policy support for SRM is significantly lower vis-à-vis ecosystem-based CDR, $U{=}260.00$, $Z{=}2.321$, $p{=}.020$, and engineered CDR, $U{=}255.50$, $Z{=}2.190$, $p{=}.027$; support for

the two types of CDR does not differ, U=175.00, Z= -0.161, p=.885. The latter results are reported in Supplementary Table 5a.

Supplementary Table 5a: Policy Support for Global North sample, by technology category

	SRM	Nature-based CDR	Engineered CDR
Independent national government policies restricting use of technology	26.74%[1,a] (4.55%)	24.91%[1,a] (6.38%)	26.14%[1,a] (5.50%)
National-level support and funding by governments to enable technology for public and private research and development	44.52%[1,a] (8.42%)	62.11%[1,b] (7.88%)	53.75%[1,c] (6.14%)
Information and engagement campaigns to consult and inform public	49.62%[1,a] (6.38%)	53.13%[1,a] (5.21%)	51.96%[1,a] (6.01%)
International ban or moratorium on technologies deemed risky	35.49%[1,a] (5.97%)	34.06%[1,a] (7.97%)	35.22%[1,a] (5.92%)
Establishment of international organization to conduct oversight and set standards	43.20%[1,a] (3.78%)	50.82%[1,b] (5.61%)	47.40%[1,b] (4.11%)
Creation of international scientific agency to explore and start testing and development	44.11%[1] (4.32%)	.	.
Global-level market for trading carbon credits and/or offsets	.	26.49%[1,a] (6.41%)	24.23%[1,a] (5.80%)
Generating special report at international level (e.g., by IPCC) to evaluate and assess technologies	40.18%[1,a] (5.58%)	44.99%[1,b] (5.04%)	44.50%[1,b] (4.48%)
None of the above	12.44%[1,a] (4.90%)	9.43%[1,a] (3.84%)	11.97%[1,a] (4.47%)

Note: N=30,284 participants. Standard errors in parentheses. If different letters present for columns of given row ("a" vs. "b" or "c"), significant differences exist regarding support for the specific policy across the respective technology categories ($p < 0.05$), according to pairwise, independent-samples (two-tailed) Kruskal-Wallis H testing, with significance values adjusted by Bonferroni correction for multiple tests. If letters are the same (e.g., all categories marked by "a"), no significant differences exist. If numbers are different between respective cells in Supplementary Tables 5a and 5b (i.e., "2" appears in corresponding cell in Supplementary Table 5b), significant differences exist between support for a given policy in Global North and Global South, according to pairwise, independent-samples Mann-Whitney U testing. Means (rather than mean ranks, on which the tests are based) are reported.

Turning to the Global South cohort, as represented in Supplementary Table 5b in the main text, policy support does not significantly differ across the technology types for "independent national government policies restricting use of technology", $\chi2(2) = 1.328$, p= .515, "information and engagement campaigns to consult and inform public", $\chi2(2) = 0.683$, p= .711, "international ban or moratorium on technologies deemed risky", $\chi2(2) = 1.324$, p= .516, "establishment of international organization to conduct oversight and set standards", $\chi2(2)$ =0.582, p=.748, "global-level market for trading carbon credits and/or offsets", $\chi2(2)$ = 0.182, p= .699, "generating special report at international level (e.g., by IPCC) to evaluate and assess technologies", $\chi2(2) = 2.791$, p= .248, and "none of the above, $\chi2(2) = 0.651$, p = .722. In fact, there are significant differences between technology types only in terms of policy support for "national-level support and funding by governments to enable technology for public and private research and development", $\chi2(2) = 8.670$, p=.013. Looking at pairwise comparisons, support for this policy is significantly lower for SRM vis-à-vis ecosystem-based

CDR, *H* = -12.136, *Z*= -2.944, *p* =.010, while there is no difference in terms of policy support for SRM vis-à-vis engineered CDR, *H* = -6.136, *Z*= -1.489, *p* =.410, ecosystem-based CDR vis-à-vis engineered CDR, *H* = 6.000, *Z*=1.456, *p* =.436.

Supplementary Table 5b: Policy Support for Global South sample, by technology category

	SRM	Nature-based CDR	Engineered CDR
Independent national government policies restricting use of technology	34.30%[2,a] (7.17%)	33.15%[2,a] (8.21%)	35.97%[2,a] (7.52%)
National-level support and funding by governments to enable technology for public and private research and development	60.85%[2,a] (5.30%)	67.66%[2,b] (4.78%)	64.80%[2,ab] (4.98%)
Information and engagement campaigns to consult and inform public	60.85%[2,a] (7.61%)	62.86%[2,a] (8.20%)	60.42%[2,a] (8.55%)
International ban or moratorium on technologies deemed risky	33.58%[1,a] (4.65%)	34.35%[1,a] (7.10%)	35.95%[1,a] (6.60%)
Establishment of international organization to conduct oversight and set standards	54.31%[2,a] (7.83%)	56.69%[1,a] (9.15%)	56.04%[2,a] (9.87%)
Creation of international scientific agency to explore and start testing and development	53.93%[2] (6.00%)	.	.
Global-level market for trading carbon credits and/or offsets	.	35.80%[2,a] (5.08%)	34.85%[2,a] (6.17%)
Generating special report at international level (e.g., by IPCC) to evaluate and assess technologies	53.22%[2,a] (2.99%)	56.89%[2,a] (4.95%)	55.06%[2,a] (6.28%)
None of the above	3.05%[2,a] (1.69%)	3.35%[2,a] (1.63%)	3.67%[2,a] (2.17%)

Note: N=30,284 participants. Standard errors in parentheses. If different letters present for columns of given row ("a" vs. "b"), this indicates that significant differences exist regarding support for the specific policy across the respective technology categories ($p < 0.05$), according to pairwise, independent-samples (two-tailed) Kruskal-Wallis *H* testing, with significance values adjusted by using Bonferroni correction for multiple tests. If letters are the same (e.g., all categories marked by "a"), no significant differences exist. If two letters appear (e.g., "ab"), as for "engineered CDR" in the second row of Table 5b, this indicates that support for this policy does not significantly differ from those marked by "a" or "b", though these do differ from one another. If the numbers are different between the respective cells in Tables 5a and 5b (i.e., "2" appears in corresponding cell in Table 5b), this indicates that significant differences exist between support for a given policy in Global North and Global South, according to pairwise, independent-samples Mann-Whitney U testing. Means (rather than mean ranks, on which the tests are based) are reported.

Finally, we employed non-parametric independent-samples (two-tailed) Mann-Whitney *U* testing to make comparisons between the Global North and Global South cohorts, doing so for each of the technology types. For SRM, we find statistically greater policy support in the Global South for "independent national government policies restricting use of technology", *U*=35.50, *Z*= -2.970, *p*=.002, "national-level support and funding by governments to enable technology for public and private research and development", *U*=11.50, *Z*= -4.004, *p* =.000, "information and engagement campaigns to consult and inform public", *U*=28.00, *Z*= -3.293, *p* =.001, "establishment of international organization to conduct oversight and set standards", *U*=20.50, *Z*= -3.615, *p* =.000, "creation of international scientific agency to explore and start testing and development", *U*=19.00, *Z*= -3.680, *p* =.000, and "generating special report at international level (e.g., by IPCC) to evaluate and assess technologies", *U*=0.00, *Z*= -4.498, *p*

=.000, while those in the Global North were significantly more likely to select "none of the above, U=205.50, Z= 4.348, p =.000. Conversely, the two cohorts did not differ in their support for an "international ban or moratorium on technologies deemed risky", U=129.00, Z= 1.055, p =.307. For ecosystem-based CDR, we find statistically greater policy support in the Global South for "independent national government policies restricting use of technology", U=43.00, Z= -2.647, p=.007, "national-level support and funding by governments to enable technology for public and private research and development", U=58.00, Z= -2.002, p =.047, "information and engagement campaigns to consult and inform public", U=37.00, Z= -2.905, p =.003, "global-level market for trading carbon credits and/or offsets", U=28.00, Z= -3.293, p =.001, and "generating special report at international level (e.g., by IPCC) to evaluate and assess technologies", U=9.50, Z= -4.089, p =.000, while those in the Global North were significantly more likely to select "none of the above, U=199.00, Z= 4.068, p =.000. Conversely, the two cohorts did not differ in their support for an "international ban or moratorium on technologies deemed risky", U=99.50, Z= -0.215, p =.832, or "establishment of international organization to conduct oversight and set standards", U=64.00, Z= -1.743, p =.085. Lastly, for engineered CDR, we find statistically greater policy support in the Global South for "independent national government policies restricting use of technology", U=27.00, Z= -3.336, p=.000, "national-level support and funding by governments to enable technology for public and private research and development", U=11.00, Z= -4.025, p =.000, "information and engagement campaigns to consult and inform public", U=46.50, Z= -2.497, p =.011, "establishment of international organization to conduct oversight and set standards", U=44.00, Z= -2.605, p =.008, "global-level market for trading carbon credits and/or offsets", U=22.00, Z= -3.551, p =.000, and "generating special report at international level (e.g., by IPCC) to evaluate and assess technologies", U=21.50, Z= -3.572, p =.000, while those in the Global North were significantly more likely to select "none of the above, U=200.50, Z= 4.132, p =.000. Conversely, the two cohorts did not differ in their support for an "international ban or moratorium on technologies deemed risky", U=97.00, Z= -0.323, p =.767.

Hierarchical Linear Regression Analysis Outputs

Supplementary Figure 3: Model Summary and Coefficient Estimates for Hierarchical Linear Regression Analysis of Support for Technology

Model Summary

					Change Statistics				
Model	R	R Square	Adjusted R Square	Std. Error of the Estimate	R Square Change	F Change	df1	df2	Sig. F Change
1	,679[a]	,462	,458	,33187	,462	127,364	2	297	<,001
2	,701[b]	,492	,487	,32300	,030	17,528	1	296	<,001
3	,703[c]	,494	,484	,32382	,003	,502	3	293	,681

a. Predictors: (Constant), Dummy for Global North, Global South, Dummy for SRM

b. Predictors: (Constant), Dummy for Global North, Global South, Dummy for SRM, Age (mean)

c. Predictors: (Constant), Dummy for Global North, Global South, Dummy for SRM, Age (mean), Q13 - Worried about Climate Change, Personal Experience with Major Natural Disaster, Personal Harm from Climate Change

Coefficients[a]

		Unstandardized Coefficients		Standardized Coefficients		
Model		B	Std. Error	Beta	t	Sig.
1	(Constant)	4,180	,034		122,818	<,001
	Dummy for SRM	-,525	,042	-,535	-12,557	<,001
	Dummy for Global North, Global South	-,392	,040	-,419	-9,852	<,001
2	(Constant)	5,557	,330		16,816	<,001
	Dummy for SRM	-,525	,041	-,535	-12,902	<,001
	Dummy for Global North, Global South	-,090	,082	-,096	-1,098	,273
	Age (mean)	-,037	,009	-,367	-4,187	<,001
3	(Constant)	5,663	,550		10,297	<,001
	Dummy for SRM	-,525	,041	-,535	-12,869	<,001
	Dummy for Global North, Global South	-,097	,085	-,104	-1,149	,251
	Age (mean)	-,038	,011	-,376	-3,401	<,001
	Personal Experience with Major Natural Disaster	,127	,200	,047	,637	,525
	Personal Harm from Climate Change	-,181	,157	-,145	-1,151	,251
	Q13 - Worried about Climate Change	,110	,138	,088	,797	,426

a. Dependent Variable: Q6 - Overall Support

Supplementary Figure 4: Nonparametric Correlations between Age(mean) and Climate Change Beliefs

Correlations

		Age (mean)	Concern over climate change	Personal harm from climate change	Personal experience with major natural disaster	Science and technology as solution to climate change
Age (mean)	Correlation Coefficient	1,000	-,587**	-,731**	-,778**	-,687**
	Sig. (2-tailed)	.	<,001	<,001	<,001	<,001
	N	300	300	300	300	300
Concern over climate change	Correlation Coefficient	-,587**	1,000	,908**	,697**	,473**
	Sig. (2-tailed)	<,001	.	<,001	<,001	<,001
	N	300	300	300	300	300
Personal harm from climate change	Correlation Coefficient	-,731**	,908**	1,000	,760**	,516**
	Sig. (2-tailed)	<,001	<,001	.	<,001	<,001
	N	300	300	300	300	300
Personal experience with major natural disaster	Correlation Coefficient	-,778**	,697**	,760**	1,000	,487**
	Sig. (2-tailed)	<,001	<,001	<,001	.	<,001
	N	300	300	300	300	300
Science and technology as solution to climate change	Correlation Coefficient	-,687**	,473**	,516**	,487**	1,000
	Sig. (2-tailed)	<,001	<,001	<,001	<,001	.
	N	300	300	300	300	300

**. Correlation is significant at the 0.01 level (2-tailed).

Note: Correlation coefficients refer to *Spearman's ρ*, which is a nonparametric measure of rank correlation.

Supplementary Figure 5: Scatter plots of technology support in terms of age (mean), by technology category (1-5 scale: 1= Strictly reject; 3=Neither support nor reject; 5=Fully support)

Solar Radiation Modification (SRM)

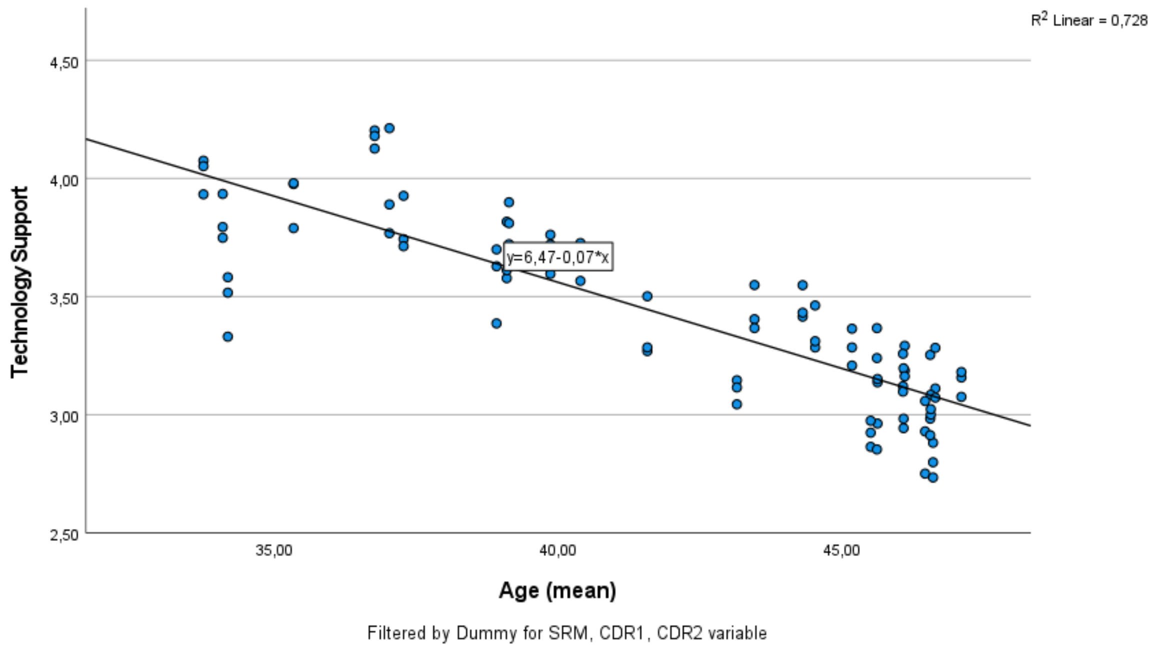

Engineered Carbon Dioxide Removal (CDR1)

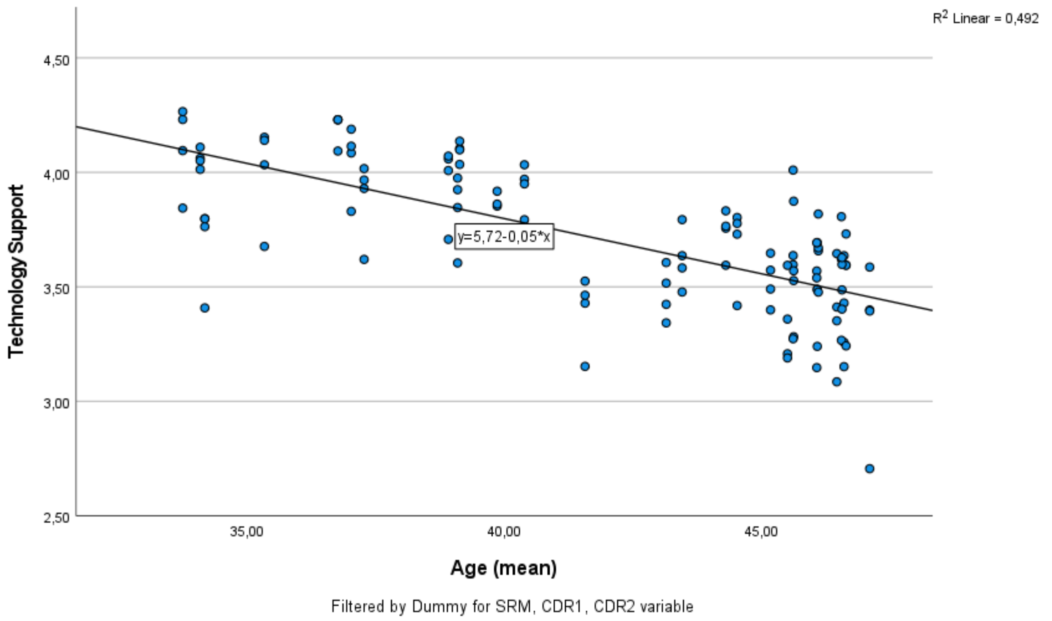

Ecosystem-based Carbon Dioxide Removal (CDR2)

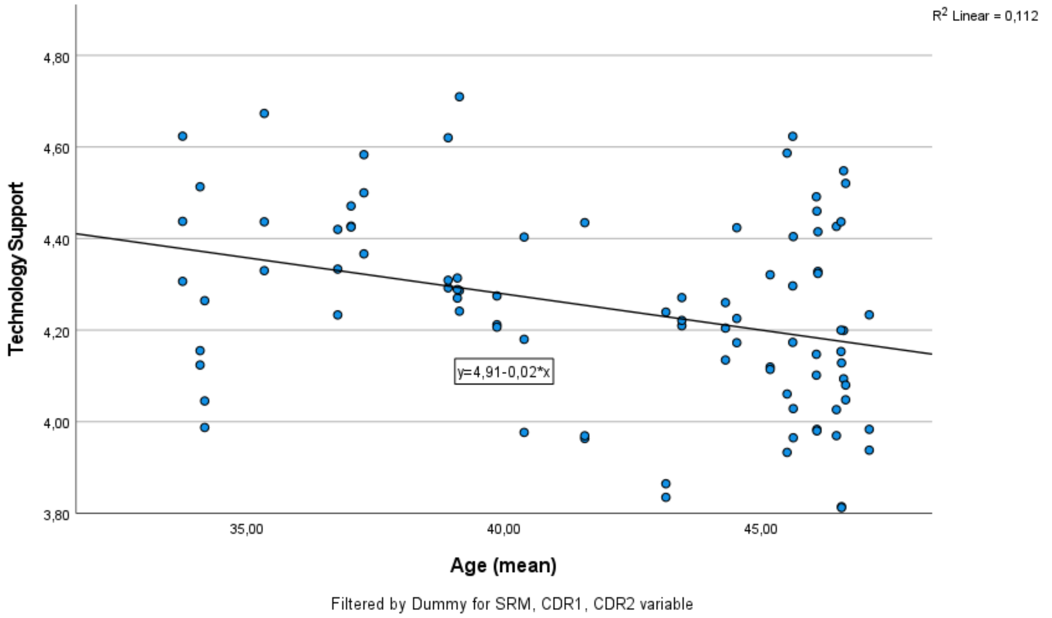

Source: Authors. Scatter plots drawn using SPSS, with line of fit imposed to assist interpretation. *Support* represents overall support for technology, across activities for research, small-scale field trials, and broad deployment. These items were combined by taking their average, given the high levels of correlation between them (i.e., the lowest Spearman's rho correlation was 0.956 between small-scale field trials and broader deployment). Responses were on a five-point scale, with options of: 1=Strictly reject; 2=Somewhat reject; 3=Neither reject nor support; 4= Somewhat support; 5= Fully support; along with a "Don't know" option (the latter was coded as a missing value). Values for R^2 are included in upper right to give a sense of the explanatory power of (mean) age for each technology category.

Supplementary Information References

Armstrong, R.A. (2014). When to use the Bonferroni correction. *Ophthalmic and Physiological Optics*, *34*(5), 502-508.

Feldman, L., & Hart, P. S. (2021). Upping the ante? The effects of "emergency" and "crisis" framing in climate change news. *Climatic Change*, *169*(1-2), 10.

Jobin, M., & Siegrist, M. (2020). Support for the Deployment of Climate Engineering: A Comparison of Ten Different Technologies. *Risk Analysis*, *40*(5), 1058–1078. https://doi.org/10.1111/risa.13462

Pidgeon, N. F., & Spence, E. (2017). Perceptions of enhanced weathering as a biological negative emissions option. *Biology Letters*, *13*(4), 20170024. https://doi.org/10.1098/rsbl.2017.0024

Rothman, K.J. (1990). No adjustments are needed for multiple comparisons. *Epidemiology*, 43-46.

Steentjes, K., Pidgeon, N., Poortinga, W., Corner, A., Arnold, A., Böhm, G., Mays, C., Poumadère, M., Ruddat, M., Scheer, D., Sonnberger, M., Tvinnereim, E. (2017). European Perceptions of Climate Change: Topline findings of a survey conducted in four European countries in 2016. Cardiff, UK: Cardiff University

Van der Werff, E., Steg, L., & Keizer, K. (2013). The value of environmental self-identity: The relationship between biospheric values, environmental self-identity and environmental preferences, intentions and behaviour. *Journal of Environmental Psychology*, *34*, 55-63.

Wolske, K. S., Raimi, K. T., Campbell-Arvai, V., & Hart, P. S. (2019). Public support for carbon dioxide removal strategies: The role of tampering with nature perceptions. *Climatic Change*, *152*(3), 345–361. https://doi.org/10.1007/s10584-019-02375-z

Wright, M. J., Teagle, D. A. H., & Feetham, P. M. (2014). A quantitative evaluation of the public response to climate engineering. *Nature Climate Change*, *4*(2), 106-110. https://doi.org/10.1038/nclimate2087

Survey preparation and Quality controls

Languages and Translation

The survey and all related information provided to participants were translated (by professional translators) into the language(s) most predominantly spoken in target countries. In total, this entailed the survey being translated into a total of 19 languages. For countries which are deeply multilingual and where many languages were spoken by the different constituent ethnic groups (e.g., India, Kenya, Nigeria), we employed the dominant *lingua franca*, typically English. After soft launch of the survey in these countries, examination of the sample composition as well as a check of individual comments in available open-ended questions satisfactorily ruled out use of English presenting any difficulties. In India, however, soft launch revealed that reliance on English resulted in apparent restrictions on which individuals could take part. Data collection was thus paused to allow for all materials to be translated to Hindi, with the survey then opened back up and individuals allowed to choose whether they wanted to take part in English or Hindi. A similar choice was given to participants in five other countries where (at least) two languages were spoken by large subsets of the population, to encourage their participation in the language that best suited them – for instance, those in the United States could choose between Spanish and English, those in Canada between English and French, and those in Switzerland between Italian, French, and German (Supplementary Table 1). All of this was intended to facilitate as broad and deep of participation in the survey (and topic) as possible. Furthermore, in view of sensitivities around certain questions in some countries, all questions about educational attainment, income levels, and so on were subjected to processes of "localization" before being launched. This was done with strong appreciation of how words, concepts, and even emotions are expressed in the Spanish of Chile or Dominican Republic is different from that in the Spanish of Spain. In this manner – also in the case of the countries in which the survey was presented in German, French, Italian, and Chinese Simplified – this ensured surveys would closely reflect the language used in the local context, and thereby avoid any confusion or uncertainty being introduced.

On the translation process, all materials were translated and proofread by separate professional translators who are native in the respective language and for all countries, before undergoing an additional review by another linguist along with "quality assurance" by internal teams at the translation agencies. At Norstat, there was an additional check, where available, by an internal native speaker who checks the accuracy of translation once more. This is then followed by two more project managers (or executives) reviewing to ensure all the overlays have been correctly applied and generally ensuring overall accuracy. As a final step, one of the co-authors carried out trial runs with both survey versions (mobile and desktop) for all languages, with this being done by a co-author native in the language if possible or, at a minimum, employed translation tools. In total, survey translations were screened and pre-tested by four or five, (and often more) individuals, from the research team and at Norstat. Such checks were conducted (i) for desktop and mobile versions to ensure there were no issues with formatting of overlays, i.e., to avoid issues, especially for "longer" languages such as German or Japanese, of text not fitting into boxes. These checks (ii) also involved inspection of the translations, e.g., to ensure there were no inconsistencies with how specific technologies or concepts were described in the survey and to confirm that

professional translators employed the suggested keyword translations from the native-language experts. Given the specialist nature of the topic – and possible concerns certain words may be difficult for those without expertise in the field to translate – a list of 21 keywords (e.g., geoengineering, carbon sequestration, climate mitigation, direct air capture) was prepared for a network of native-language experts who worked in the field to translate. The list, along with any additional comments, were forwarded to translators to facilitate and guide translation activities. In general, this underscores the difficulty of translating potentially novel terms that are not widely known or wholly unknown in the target language – and which may thus have to assume a "transliterated" form, i.e., by translating the words without trying to fit them into the context of the language itself – along with the importance of engaging with local experts, where available. In cases where there was a divergence between the translations of the topic experts and the professional translators, e.g., for French, Danish, and Norwegian, this was subjected to back-and-forth discussion over why a decision was taken and thereby resolved. As an example, translation of "deployment" in Danish and Norwegian was discussed, and ultimately changed, to better reflect the initial understanding in English rather than the sense of "application" (in Danish: "Udrulning" versus "Udbredelsen"). There were similar situations in French leading to, e.g., changing one of the items for the *aversion to tampering with nature* scale so that it was described as a "downfall" (*chute*) rather than a "loss" (*perte*). In the case of Chile (Spanish), the translation of one of the items on risks and benefits was initially translated to convey it "… can be safe if done in a controlled way" rather than "can be done safely in a controlled fashion." Accordingly, this was changed in Spanish to: "… se puede hacer de forma segura y controlada". Furthermore, there were extensive revisions to the German translations, which were resolved through back-and-forth discussions the translators to ensure texts were comprehensible, easy to read, and moreover aligned with the English originals. By way of an example, for "marine cloud brightening", a description was changed from "…in order to make clouds *whiter*" to "… in order to make clouds *brighter*" (i.e., "…um die Wolken *weißer zu machen*" to "... um die Wolken *aufzuhellen*"), Similarly, the translation for "enhanced weathering" was changed from "*verbesserte* Verwitterung" (i.e., *improved* weathering) to "*beschleunigte* Verwittering" (i.e., *accelerated* weathering). In this vein, where disagreement specifically involved translations of technologies, we searched to see whether one version already appeared in the target language, in which case this one would be used. In general, we also tended to include the English term in parentheses next to the translation, with this also suggested by many of the topic experts. Overall, the steps undertaken underscore the efforts made to achieve the best possible translation.

Quality Checks and Participant Replacement

Multiple steps were taken to ensure the final dataset was of high quality, specifically through several quality checks and, ultimately, by removing and replacing respondents which failed to satisfy a range of criteria. First, there were two comprehension checks though, based on results from the soft launch and given unfamiliarity of the topic, we opted not to exclude participants that failed to answer the question correctly in two attempts. Conversely, if someone answered the second (true/false) question wrong twice, then were removed, taking this as a good indicator of lack of attention. Second, there were two trap questions included in the survey, e.g., where participants were instructed to select "Strongly agree" – if individuals

answered both questions incorrectly, they were also removed. Ultimately, we opted against removing participants if they only answered one trap question wrong given that the survey entailed lots of novel information, which also could be quite complex. Third, a “speeder flag” was instituted whereby participants who completed the survey in less than one-third of the median length of survey for a particular country were removed – in addition, for countries where the median length of survey was quite low, i.e., less than seven minutes, such individuals were also often removed and replaced, given questions of how realistic such a completion time would be given the minimum time constraints imposed for reading the information texts. Fourth, an open-ended question was included at the end of the survey, both to inquire about any possible problems and thereby make improvements to the survey and, in the case of problematic answers, as a reason to exclude participants. Lastly, at an administrative level, those with duplicate IP addresses and/or geolocation data which was, for whatever reason, not valid for a specific country.

In addition, all countries underwent a soft launch with 5-10% of the full sample to identify any potential issues, including with the translations, programming, and localization utilized to tailor the survey to the country context. These checks also considered whether changes were needed, e.g., to levels for income and education. Also, especially in view of the importance of issues of gender identity, we paid close attention to how the question about one’s gender was framed in the languages. A handful of initial translations (e.g., Swedish, Norwegian’) did not ask about gender but rather something to the effect of “Are you a man or a woman?”. Translations were thus changed to a more neutral form, i.e., “What is your gender?”. Similarly, in languages (like, German) where certain actors (e.g., climate activists or neighbors) can have both a masculine and feminine form, we made sure to include both of these in the answer option. In addition, as part of the soft-launch process, the data for every single country was assessed to make sure that (a) there was sufficient heterogeneity, specifically for the questions relating to perceptions of technologies, to determine if participants may be “straightlining” responses; (b) an inordinate amount of participants were not getting the trap questions and comprehension checks incorrect – for instance, if around 45% of participants in the SL data answered one of the trap questions incorrectly – we paused data launch to confirm with Norstat that all programming was working appropriately, that there were no issues with translation that could lead to confusion (done by a native speaker in the language), and also specifically reviewed the open-ended comments of the respondents who failed this question to see if they flagged any issues; with such possibilities dismissed, and since those with incorrect answers would be “cleaned” from the final sample, we proceeded with data collection; (c) participants were not availing themselves too often for certain questions of “Prefer not to say” or “Don’t Know” or “Neither agree nor disagree”, not because the responses were themselves invalid or undesirable but, rather, to help identify if an item may not be working as expected in a given context and/or as a signal to keep an eye on something for full launch (or whether to change from an “unbalanced” to “balanced” scale; and (d) to identify early indications of the sample being unrepresentative in terms of age, gender, income, geographic region, and education. In one or two extreme cases, like for income in China, this led to changes in the income levels – otherwise, a closer eye was kept on quotas and patterns in responses to given questions. One final element of the soft launch data check considered the open-ended responses to the question included at the end asking if there are any issues or problems, which doubled as a “quality check”. Almost exclusively, such responses were of a kind expressing there was “no problem” or to “thank you for the

survey”. In a couple instances, however, these helped to identify a potential problem early on, e.g., highlighting the need to use Hindi as a language for India or, more unexpectedly, to remove the question about whether one belonged to a minority group in Estonia. Upon reflection, this question seemed to be problematic for some survey participants given the Russo-Ukraine War and the presence of a sizable Russian ethnic minority group in Estonia. This question was thus removed for reasons of politically sensitivity and not wanting to irritate survey participants, as had also for instance been done for political views in China, with this question not asked in this country.
