# Supplementary material for: Public perceptions and support of climate intervention technologies across the Global North and Global South
Source: Nat Commun. 2024 Mar 6;15:2060. doi: 10.1038/s41467-024-46341-5 (PMC10918186; doi:10.1038/s41467-024-46341-5)
Supplement: Supplementary file 3 — Reporting Summary [file 41467_2024_46341_MOESM3_ESM.pdf]

Corresponding author(s): Chad M. Baum, NCOMMS-23-26418

Last updated by author(s): Dec 22, 2023

Reporting Summary

Nature Portfolio wishes to improve the reproducibility of the work that we publish. This form provides structure for consistency and transparency in reporting. For further information on Nature Portfolio policies, see our Editorial Policies and the Editorial Policy Checklist.

Statistics

For all statistical analyses, confirm that the following items are present in the figure legend, table legend, main text, or Methods section.

n/a	Confirmed	
☐	☒	The exact sample size (n) for each experimental group/condition, given as a discrete number and unit of measurement
☐	☒	A statement on whether measurements were taken from distinct samples or whether the same sample was measured repeatedly
☐	☒	The statistical test(s) used AND whether they are one- or two-sided<br>*Only common tests should be described solely by name; describe more complex techniques in the Methods section.*
☐	☒	A description of all covariates tested
☐	☒	A description of any assumptions or corrections, such as tests of normality and adjustment for multiple comparisons
☐	☒	A full description of the statistical parameters including central tendency (e.g. means) or other basic estimates (e.g. regression coefficient) AND variation (e.g. standard deviation) or associated estimates of uncertainty (e.g. confidence intervals)
☐	☒	For null hypothesis testing, the test statistic (e.g. F, t, r) with confidence intervals, effect sizes, degrees of freedom and P value noted<br>*Give P values as exact values whenever suitable.*
☒	☐	For Bayesian analysis, information on the choice of priors and Markov chain Monte Carlo settings
☒	☐	For hierarchical and complex designs, identification of the appropriate level for tests and full reporting of outcomes
☐	☒	Estimates of effect sizes (e.g. Cohen's d, Pearson's r), indicating how they were calculated

Our web collection on statistics for biologists contains articles on many of the points above.

Software and code

Policy information about availability of computer code

Data collection	No such software was used.
Data analysis	Analyses were conducted using SPSS v28.0 software

For manuscripts utilizing custom algorithms or software that are central to the research but not yet described in published literature, software must be made available to editors and reviewers. We strongly encourage code deposition in a community repository (e.g. GitHub). See the Nature Portfolio guidelines for submitting code & software for further information.

Data

Policy information about availability of data

All manuscripts must include a data availability statement. This statement should provide the following information, where applicable:

- Accession codes, unique identifiers, or web links for publicly available datasets
- A description of any restrictions on data availability
- For clinical datasets or third party data, please ensure that the statement adheres to our policy

The datasets generated during and/or analyzed during the current study are not publicly available at present, both given that the peer-review process is underway and to allow for further analysis and publication of findings over the course of the project. We do however intend to make all relevant data publicly available together with the published article. At the moment, the dataset is available from the corresponding author on reasonable request, and indeed has already been privately shared on the Figshare server.

Human research participants

Policy information about studies involving human research participants and Sex and Gender in Research.

Reporting on sex and gender	As part of the quantitative survey design, we do have an item relating to sex and gender - though these results are not considered in the analysis of the current paper. In any case, participants were afforded an option of "Other/Prefer not to say".
Population characteristics	The nature of the current research is not invasive; in any case, as noted in the supplemental materials, any questions about particular data being sensitive, including those that emerged in the course of the survey(s), were handled by erring on the side of caution and not asking a question in a given market. We also ensured that participants were generally afforded an "opt-out" option should they prefer not to answer.
Recruitment	Data collection was administered online by Norstat on behalf of Aarhus University using quota sampling, with informed consent obtained from all participants included in the study, all of whom were notified in advance of how their data would be handled as well as their right to withdraw at any time, and with all data delivered to researchers de-identified and anonymized by Norstat. Surveys were nationally representative of country populations 18-74 in terms of age gender, and geographic region along with broad quotas for education and income. Upon successful completion of the survey, participants received monetary compensation directly from the professional survey firm, Norstat.
Ethics oversight	All components of the research were granted ethical approval by relevant authorities at Aarhus University (#2021-13)

Note that full information on the approval of the study protocol must also be provided in the manuscript.

Field-specific reporting

Please select the one below that is the best fit for your research. If you are not sure, read the appropriate sections before making your selection.

☐ Life sciences ☒ Behavioural & social sciences ☐ Ecological, evolutionary & environmental sciences

For a reference copy of the document with all sections, see nature.com/documents/nr-reporting-summary-flat.pdf

Behavioural & social sciences study design

All studies must disclose on these points even when the disclosure is negative.

Study description	Quantitative study which uses nationally representative data from a large-scale, cross-country survey exercise taking place in 30 countries and 19 languages around the world, including in both the Global North and Global South, to assess public perceptions of climate intervention technologies (i.e., solar radiation management and carbon dioxide removal).
Research sample	The total sample consisted of 30,284 participants with at least 1,000 in each of the 30 countries surveyed. Surveys were nationally representative of country populations 18-74 in terms of age gender, and geographic region along with broad quotas for education and income. This approach was undertaken to ensure that the survey results, as much as possible, reflected perceptions of the countries in question, e.g., avoiding a bias towards the wealthiest or most highly educated. The only departures from national representativeness were: in Singapore, for those 55-74; in Greece, Switzerland, India, Dominican Republic, Chile, India, Saudi Arabia, Indonesia, for those 65-74; in Saudi Arabia, Indonesia, Chile, for some living in smaller regions; and in the United Kingdom, for those in North East England. Demographic characteristics for countries are available on request from authors.
Sampling strategy	Data collection was administered online by Norstat on behalf of Aarhus University using quota sampling to ensure national representativeness of country populations 18-74 in terms of age gender, and geographic region along with broad quotas for education and income. Given the novelty of the topic and the fact that many of the countries had never been surveyed in this context before, we aimed to have as large of a sample as possible while also achieving a broad scope in terms of countries included. In addition, given that participants would be assigned to one of three groups, more than 300 participants would be needed to have a power of 0.70-0.80 for a Cohen's d effect size which is small in nature (i.e., 0.2).
Data collection	Data collection employed an online survey design administed by Norstat and consisting of randomized assignment to one of the three technology types (solar radiation management, nature-based carbon removal, engineered carbon removal) and measures related to risks and benefits, support for different types of activities, support for various policy options along with questions on sociodemographic characteristics, beliefs about climate change and environment, and trust in institutions and actors and credibility of sources of information. Survey design, especially the information texts that were provided to participants, was revised and finalized by extensive piloting, input from external researchers, professional translators, and programmers and survey experts at Norstat, and drawing on the of results a soft launch undertaken for each country. Upon successful completion of the survey, participants received monetary compensation directly from the professional survey firm, Norstat.
Timing	Data collection for the surveys proceeded in stages for the thirty countries from August to December 2022.
Data exclusions	No data was excluded from the current study.

Non-participation	Participants were told, at the stage of providing full and informed consent before the beginning of the study, that they had the right to withdraw their participation at any time, or indeed not to proceed with the survey. As only completed responses were provided to researchers by the professional survey firm in a de-identified and anonymized form, we are not aware of those who might have chosen not to participate, for whatever reason.
Randomization	Participants were randomly assigned to provide responses related to one of the three technology types: solar radiation management, nature-based carbon dioxide removal, and engineered carbon dioxide removal. This assignment was done in a purely randomized manner by the professional survey firm, Norstat.

Reporting for specific materials, systems and methods

We require information from authors about some types of materials, experimental systems and methods used in many studies. Here, indicate whether each material, system or method listed is relevant to your study. If you are not sure if a list item applies to your research, read the appropriate section before selecting a response.

Materials & experimental systems

n/a	Involved in the study
☒	☐ Antibodies
☒	☐ Eukaryotic cell lines
☒	☐ Palaeontology and archaeology
☒	☐ Animals and other organisms
☒	☐ Clinical data
☒	☐ Dual use research of concern

Methods

n/a	Involved in the study
☒	☐ ChIP-seq
☒	☐ Flow cytometry
☒	☐ MRI-based neuroimaging
